# Effect of Mono and Di-rhamnolipids on Biofilms Pre-formed by *Bacillus subtilis* BBK006

**DOI:** 10.1007/s00284-016-1046-4

**Published:** 2016-04-25

**Authors:** Mayri A. Díaz De Rienzo, Peter J. Martin

**Affiliations:** School of Chemical Engineering and Analytical Science, The University of Manchester, Manchester, UK

## Abstract

Different microbial inhibition strategies based on the planktonic bacterial physiology have been known to have limited efficacy on the growth of biofilms communities. This problem can be exacerbated by the emergence of increasingly resistant clinical strains. Biosurfactants have merited renewed interest in both clinical and hygienic sectors due to their potential to disperse microbial biofilms. In this work, we explore the aspects of *Bacillus subtilis* BBK006 biofilms and examine the contribution of biologically derived surface-active agents (rhamnolipids) to the disruption or inhibition of microbial biofilms produced by *Bacillus subtilis* BBK006. The ability of mono-rhamnolipids (Rha–C_10_–C_10_) produced by *Pseudomonas aeruginosa* ATCC 9027 and the di-rhamnolipids (Rha–Rha–C_14_–C_14_) produced by *Burkholderia thailandensis* E264, and phosphate-buffered saline to disrupt biofilm of *Bacillus subtilis* BBK006 was evaluated. The biofilm produced by *Bacillus subtilis* BBK006 was more sensitive to the di-rhamnolipids (0.4 g/L) produced by *Burkholderia thailandensis* than the mono-rhamnolipids (0.4 g/L) produced by *Pseudomonas aeruginosa* ATCC 9027. Rhamnolipids are biologically produced compounds safe for human use. This makes them ideal candidates for use in new generations of bacterial dispersal agents and useful for use as adjuvants for existing microbial suppression or eradication strategies.

## Introduction

Biofilms are communities of surface-associated microbial cells enclosed in an extracellular polymeric substance (EPS) matrix. Microbial biofilms represent a different bacterial physiology constituted by a multicellular phenotype which is (generally) very different from planktonic bacteria. Biofilms have been implicated in chronic infections [9]. In the biofilm physiology, these pathogens are several orders of magnitude more resistant to disruption (or killing) by antibiotics than their planktonic counterparts of the same species [12, 13, 15]. The recent advances on biofilm research have enabled researchers to develop more effective bacterial inhibition strategies; currently, there are two main ones [3]: the first is based on the formulation of new antibiofilm molecules and the second the construction of biofilm-resistant surfaces [18].

Biosurfactants are amphiphilic compounds produced on living surfaces, mostly on microbial cells [16]. Biosurfactants have long been reported as molecules with several applications in the industry: detergents, textiles, and with potential applications in environmental and biomedical related areas [8], and more recently as promising candidates for the inhibition of microbial biofilms with anti-adhesive and disruptors properties [7]. Rhamnolipid is a glycolipid biosurfactant constituted of di- or mono-rhamnose sugars attached to a fatty acid chain. These biosurfactants were previously reported as antibacterial agents against *S. aureus*, *Bacillus sp*, and *Klebsiella pneumoniae* [4, 7, 8, 11]. One of the hypotheses proposed for the biofilm inhibition by rhamnolipids is that they could be involved in the removal of extracellular polymeric substances (EPS) and destruction of microcolonies altering the biofilm environment by their surface activity.

Rhamnolipids were originally isolated from *P. aeruginosa*, analogues were also produced by isolates of *Burkholderia thailandensis* [10, 21], which has increased the research interest due to its non-pathogenic nature. In this work, we explore the ability of mono-rhamnolipids (Rha–C_10_–C_10_) produced by *Pseudomonas aeruginosa* ATCC 9027 and the di-rhamnolipids (Rha–Rha–C_14_–C_14_) produced by *Burkholderia thailandensis* to disrupt or inhibit microbial biofilms produced by *Bacillus subtilis* BBK006.

## Materials and Methods

### Microorganisms and Media

*P. aeruginosa* ATCC 9027 and *B. thailandensis* E264 were maintained on nutrient agar slants at 4 °C in order to minimize biological activity, and were subcultured every month. Each slant was used to obtain a bacterial suspension, with the optical density (570 nm) adjusted to give 10^7^ CFU/mL for each of the strains used. The standard medium for the production of rhamnolipids by *P. aeruginosa* ATCC 9027 was PPGAS medium (1 g/L NH_4_Cl, 1.5 g/L KCl, 19 g/L tris–HCl, 10 g/L peptone, and 0.1 g/LMgSO_4_·7H_2_O) at pH 7.4. The fermentation medium contained the same growth medium, with glucose (0.5 %), as a carbon source. For the production of rhamnolipids by *B. thailandensis* E264 the media used was nutrient broth (NB) (8 g/L), with glycerol (20 g/L). For the antimicrobial assays *Bacillus subtilis* BBK006 was stored in nutrient broth plus 20 % glycerol at −80 °C, and used when needed.

### Production of Rhamnolipids

Fermentation units (Electrolab FerMac 360) were used to perform batch cultivation of *P. aeruginosa* ATCC 9027 and *B. thailandensis* E264. Microorganisms used in this study were aerobically (0.5 VVM) incubated in PPGAS medium and nutrient broth, at 37 and 30 °C, respectively, at 400 rpm speed for 72 h in the case of *P. aeruginosa* ATCC 9027 and 120 h for *B. thailandensis* E264.

### Downstream Process for the Purification of Rhamnolipids

**Fig. 1:**
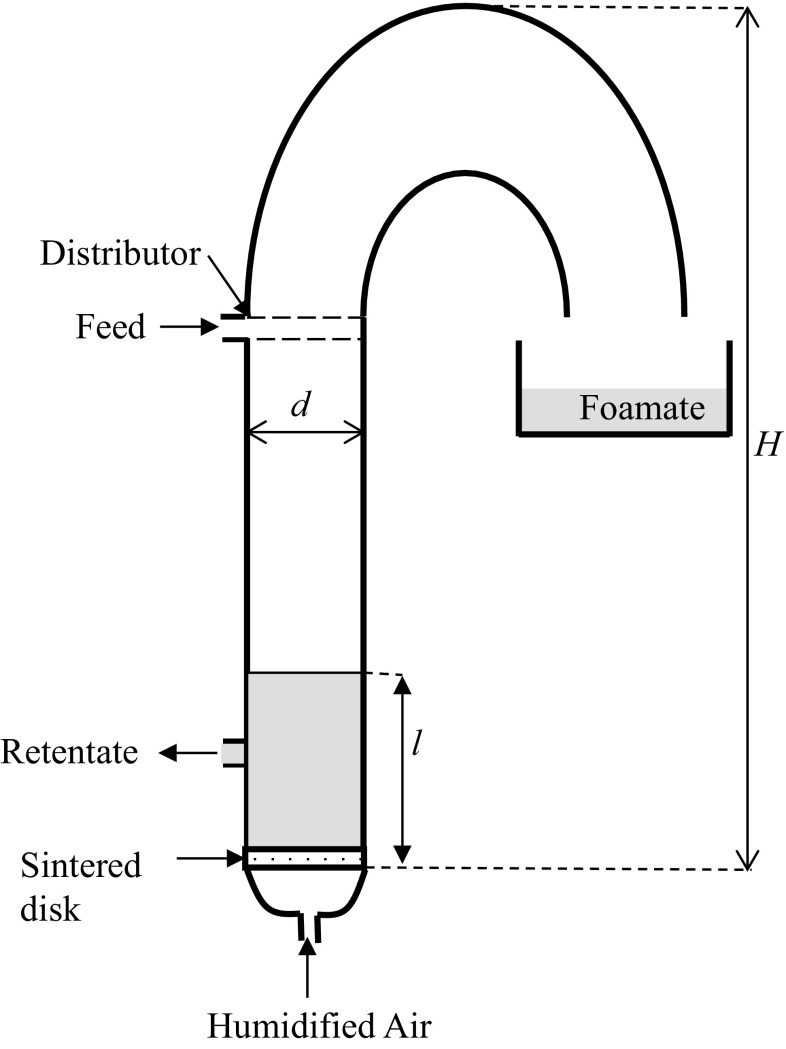
Schematic diagram of foam fractionation experimental setup

A continuous foam fractionation system in stripping mode was used as a downstream process. 4 L of rhamnolipid fermentation broth was fed into the top of the straight section of a “J”-shaped glass column of diameter, *D*, 50 mm and exposed height, *H*, 350 mm via a peristalsis pump, and a metal tube distributor at a feed flow rate of 15 mL min^−1^. Figure 1 shows the schematic diagram of the foam fractionation column [20]. Humidified air was sparged through a sintered glass disk into a liquid pool creating overflowing foam. The initial composition of the liquid pool at the bottom of the column was the same as the feed and exited the column through an exit port in such a way that a constant liquid level of 100 ± 10 mm was maintained throughout the experiment. The enriched overflowing foam was collected at the open end of the “J”-shaped section. Foam fractionation experiments were performed at different air flow rates for each microorganism. The air flow rate used for *P. aeruginosa* ATCC 9027 was 0.1 min^−1^ and for *B. thailandensis* E264 the air flow rate used was 1.2 L min^−1^. Each air flow rate was performed in duplicate with fresh fermentation broths for each foam fractionation run.

Foam fractionation was performed for 4 h to ensure steady-state conditions, and the feed, overflow, and foamate samples were collected every half an hour. The foamate samples were made air tight to prevent evaporation and placed at 4 °C overnight to collapse foam. The feed, overflow, and diluted foamate samples were analyzed for rhamnolipid concentration after the solvent extraction [17], and the product was used as the disruptor's solutions against *Bacillus subtilis* BBK006 biofilm.

### Surface Tension Measurements

Surface tension was evaluated in 10 mL aliquots of fermented cultures in the absence of biomass, using a Krüss Tensiometer K11 Mk4. Distilled water was used to calibrate the instrument and the measurements were performed in triplicate, using each culture media as a control.

### Emulsifying Capacity Determination

Emulsifying capacity was measured using 5 mL of kerosene added to 5 mL of aqueous sample. The mixture is vortex at high speed for 2 min. After 24 h, the height of the stable emulsion layer is measured. The emulsion index *E* is calculated as the ratio of the height of the emulsion layer and the total height of liquid (Eq. 1).

1 $$E = \frac{{h{\text{ emulsion}}}}{{h{\text{ total}}}} \times 100\;\%$$

### Anthrone Assay

The anthrone assay was used to estimate the concentration of the sugar moiety in the rhamnolipids, in either the free-cell culture medium (initial solution), the foamate (collapsed foam), or overflow [20]. Briefly, about 20 mg of anthrone was dissolved in a 70 % (v/v) sulfuric acid solution with gentle warming. The anthrone reaction (different concentrations were tested) was done by pipetting 0.1 mL of a test sample into an eppendorf tube. Then, 1 mL of the anthrone reagent was slowly added into the tube with agitation. After being thoroughly mixed, the tube was stoppered, and was placed in a vigorously boiling water bath for 10 min. After that, the tube was left at room temperature for 30 min. A bluish green solution was achieved, and its absorbance was measured at a wavelength of 625 nm by using a UV/Vis spectrophotometer (Shimadzu Uvmini-1240). The amount of biosurfactant in the test sample was subsequently calculated in terms of g/L of rhamnose in the test sample by using a calibration curve of the colored solution obtained from the reaction between the anthrone reagent and the standard rhamnose in the concentration range of 100–800 (g/L).

### ESI–MS Analysis

For mass analyses, partially purified rhamnolipid preparations (either the free-cell culture medium, the foamate, or overflow) were dissolved in water and characterized by ESI–MS (electrospray ionization-mass spectrometry) using a Waters LCT mass spectrometer in negative-ion mode previously tuned and calibrated on NaF. 20 μL was flow injected into a mobile phase consisting of 50 %ACN-0.1 % formic acid using a Waters Alliance 1170 HPLC.

### Growth of Biofilm in Flow Cells

Biofilms of *Bacillus subtilis* BBK006 were allowed to form in a flow cell system. The system comprised a flow cell that served as a growth chamber for the biofilms. The flow cell was supplied with nutrients and oxygen from a medium flask containing NB via a peristaltic pump (mL/h/channel) and spent medium was collected in a waste container. A bubble trapping device confined air bubbles from the tubing which otherwise could disrupt the biofilm structure in the flow cell. After 48 h of incubation at 30 °C, the medium was replaced with different treatments [phosphate-buffered saline (PBS) buffer 1X, mono-rhamnolipids 0.4 g/L, and di-rhamnolipids 0.4 g/L] for 30 min. After treatment, the cells were stained with LIVE/DEAD^®^ BacLight™ Kit and observed using a Leica SP5 inverted confocal microscope, providing highly detailed 3D information about the development of microbial biofilms using FiJi [14].

## Results and Discussion

### Production of Rhamnolipids

*Pseudomonas aeruginosa* ATCC 9027 and *Burkholderia thailandensis* E264 were able to produce glycolipids biosurfactants under aerobic conditions. After 72 h, *Pseudomonas aeruginosa* ATCC 9027 was able to produce rhamnolipids on PPGAS medium at 37 °C after 48 h, using glucose (5 g/L) as carbon source. On the other hand, *Burkholderia thailandensis* E264 was able to produce rhamnolipids on nutrient broth using glycerol (20 g/L) as carbon source.

All the rhamnolipids production in *P. aeruginosa* is associated to their virulence factors, which are regulated via quorum sensing (QS) system (nevertheless, it has not been demonstrated, yet the presence of a QS system on *B. thailandensis* is linked to the presence of *rhlA*, *rhlB*, and *rhlC*). This might be one of the reasons affecting the production yields for both rhamnolipids types (Table 1). In *Pseudomonas* case, the production is associated to the growing, while in *B. thailandensis* could be a metabolite that is been produced along with another proteins like efflux pumps and transporter, whose genes are in the *rhl* cluster.

**Table 1:** *P. aeruginosa* ATCC 9027 and *B. thailandensis* E264 biomass and rhamnolipid production yields

Microorganisms	Glycerol 20 g/L		Glucose 5 g/L	
	X (g/L)	Y_p/s_	X (g/L)	Y_p/s_
*P. aeruginosa* ATCC 9027	–	–	2.5	0.32
*B. thailandensis* E264	9.5	0.025	–	–

– Not detected

The rhamnolipids produced by *B. thailandensis* E264 reduced the surface tension up to 32 mN/m, in contrast with *P. aeruginosa* ATCC rhamnolipids where the surface tension was reduced up to 24 mN/m. This could be an indication of both molecules been structurally different, and resulting in different hydrophilic–lipophilic balance (HLB) with different values. These values are similar to those previously reported for *P. aeruginosa* sp. with values between 25 and 30 mN/m [2]. For *B. thailandensis*, the ability to produce rhamnolipids was first in 2009 [10] where the reduction of the surface tension was 42 mN/m.

The different microorganisms were assessed for their ability to form stable emulsions on the supernatant phase, and the results show a 65 % of emulsion for rhamnolipids produced by *P. aeruginosa* ATCC 9027 and a 42 % for those produced by *B. thailandensis* E264 after 11 days of cultivation.

Foam fractionation studies in continuous mode were used for the recovery of the excreted biosurfactant from the cell-free culture medium produced by both microorganisms. The molecular and surface chemistry properties of the feed, foamate, and overflow were analyzed by ESI–MS.

**Fig. 2:**
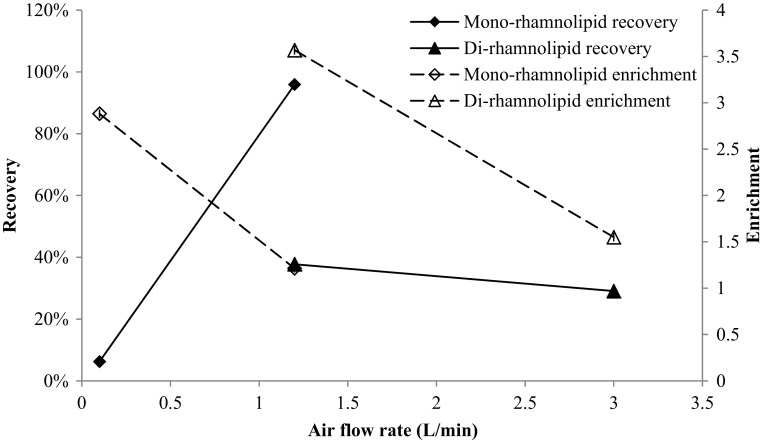
Recovery and enrichment of Rha–C_10_–C_10_ and Rha–Rha–C_14_–C_14_ with increasing air flow

Foam fractionation separation performance was evaluated in terms of recovery and enrichment. Figure 2 shows the recovery and enrichment variation with increasing air flow rate for rhamnolipids produced by *P. aeruginosa* ATCC 9027 and *B. thailandensis* E264. The results show that the recovery and enrichment of rhamnolipids produced by *P. aeruginosa* ATCC 9027 increased and decreased, respectively, with increasing air flow rate.

This is as expected for a single component system where with increasing air flow rate, the residence time of the bubbles in the column decreases. These different recovery and enrichment behaviors might suggest the presence of other surface-active species in the fermentation broth other than the rhamnolipids produced.

**Fig. 3:**
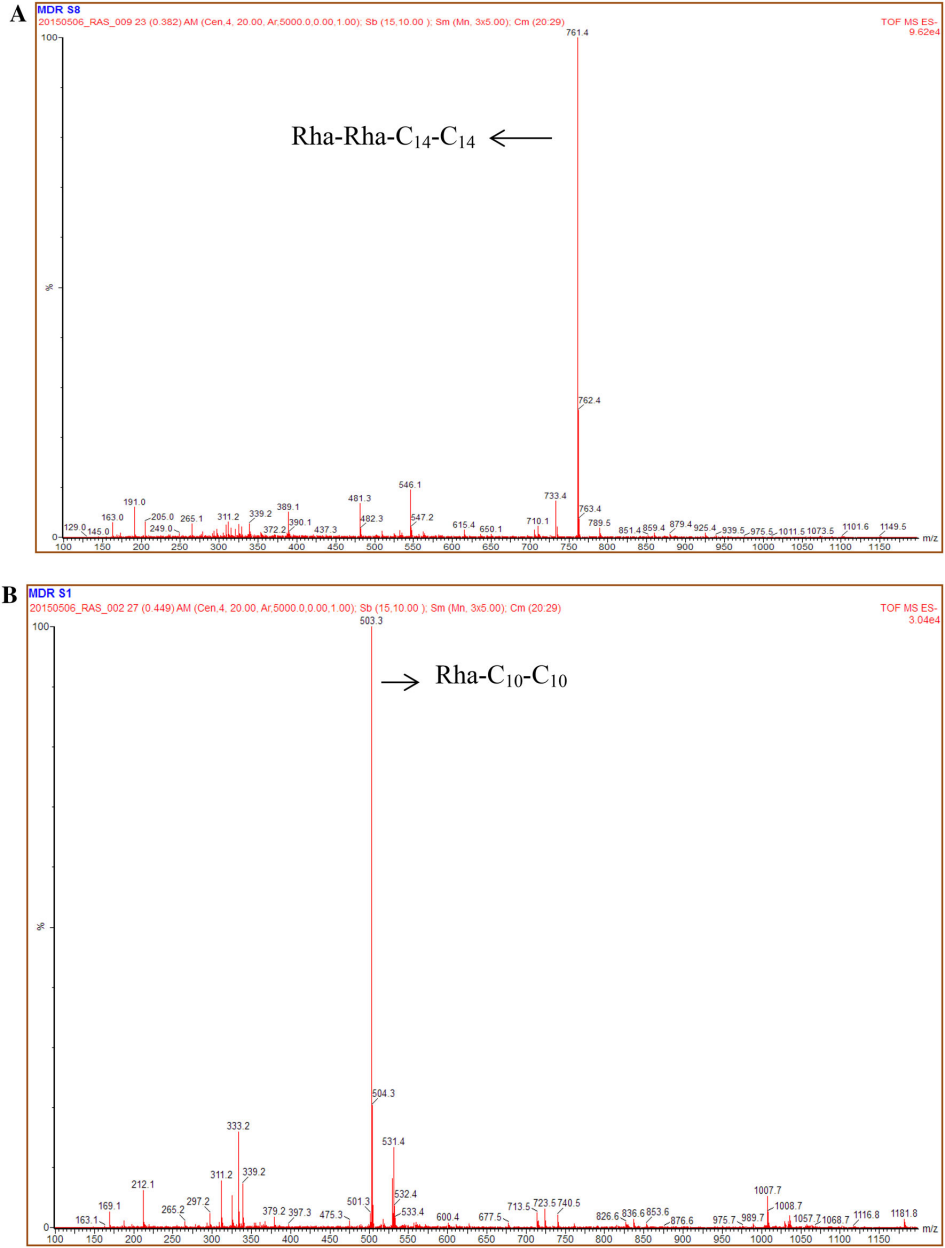
ESI-MS analysis. Spectrum of partially purified extracts from fermented cells of **a** *B. thailandensis E264* and **b** *P. aeruginosa ATCC 9027* (Rha: rhamnose molecules) in the feed fraction

ESI–MS analysis revealed the presence of different congeners of rhamnolipids produced by each microorganism. In the case of *B. thailandensis* E264, a dominant peak in the ESI–MS was shown a pseudomolecular ion of *m/z* 761 in negative-ion mode (Fig. 3a), a value that is compatible with a 2-*O*-α-l-rhamnopyranosyl-α-l-rhamnopyranosyl-β-hydroxytetradecanoyl-β-ydroxytetradecanoate (Rha–Rha–C_14_–C_14_), with a molecular weight of 762 Da, that it has been previously reported from *B. pseudomallei* and *B. plantarii and B. thailandensis* itself (6, 10).

To confirm the rhamnolipid production by *P. aeruginosa* ATCC 9027, the same ESI–MS method was used. The presence of the mono-rhamnolipid rhamnosyl-3-hydroxydecanoyl-3-hydroxydecanoate Rha–C_10_–C_10_ was revealed as a predominant peak of *m/z* 503 (Fig. 3b), in accordance with the previous reports where it has been widely studied and surveyed for the past decade [1, 16, 22].

The analysis of *B. thailandensis* E264 cultures revealed long chain rhamnolipids, with a different HLB from the one that *P. aeruginosa* ATCC 9027 was able to produce (under the conditions tested in this work), which supports the hypothesis presented where both microorganisms produce different molecules that could have different applications from a biotechnology point of view.

### Effect of Different Rhamnolipids on Pre-formed Biofilms by *Bacillus subtilis* BBK006 in Flow Cell

It has been reported before [7] that pre-formed biofilms by *Bacillus subtilis* can be disrupted by sophorolipids. In a recent work [8], studies demonstrated the effect of rhamnolipids against biofilms formed by selected gram-negative and gram-positive bacteria on static conditions; however, the effect of specific rhamnolipids congeners on biofilms formed by *Bacillus subtilis* has not been reported yet.

In this work, we evaluated the effect of Rha–C_10_–C_10_, Rha–Rha–C_14_–C_14_ and the ionic surfactant SDS on *Bacillus subtilis* BBK066 biofilms developed on a flow cell system. The cells were stained with LIVE/DEAD^®^ BacLight™ Kit, and confocal microscopy was used to analyze the data. Biofilm was grown for 2 days in continuous flow mode in a flow cell channel. Before the addition of each treatment, a developed biofilm was observed (data not shown); the thickness of the biofilm was about 15 µm. After 30 min of each treatment (PBS 1X, SDS 0.4 g/L, Rha–C_10_–C_10_ 0.4 g/L, and Rha–Rha–C_14_–C_14_ 0.4 g/L), different results were observed.

**Fig. 4:**
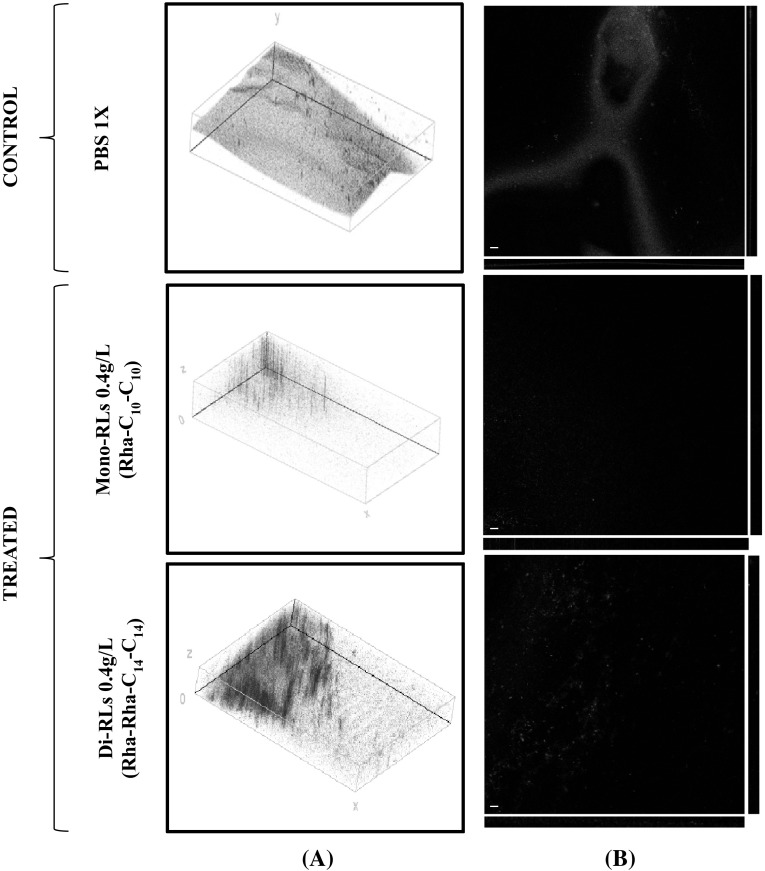
Confocal microscopy micrographs. **a** Three-dimensional (*left panels*) and **b** orthogonal reconstructions (*right panels*) of the biofilm formed by *Bacillus subtilis* BBK066. The pictures refer to the various experimental conditions as indicated on the *left*. The fluorescence is associated with live (*green*) and dead (*red*) cells, respectively. *Scale bars* represent 30 μm as indicated in micrographs (Color figure online)

The SDS has a remarkable effect on *Bacillus subtilis* BBK006 biofilm disruption at 0.04 g/L (data not shown), in comparison to those treated with PBS 1X where all the cells were well established and viable (Fig. 4). However, when the cells were treated with Rha–C_10_–C_10_ or Rha–Rha–C_14_–C_14_, the disruption is appreciated. In an interesting way, the cells treated with Rha–Rha–C_14_–C_14_ seem to be showing an inhibitory effect in the biofilm disruption judged by the red stain observed.

The results we have obtained demonstrated the inhibitory effect of rhamnolipids on pre-formed *Bacillus subtilis* BBK066 biofilms, similar to those reported by Davey [5]. In the same context, Dusane et al. [11] reported the effect of rhamnolipids on pre-formed biofilms of *Bacillus pumilus* from the marine environment, resulting in a dispersal at sub-MIC concentrations and confirming the ability to disrupt them. The effect of rhamnolipids on biofilms formed by gram-positive microorganisms relies on the ability in the removal of the matrix components to facilitate the detachment of the cells to the surface. Different congeners would possibly have different impacts on the cell surfaces where the overall charge and the length of the fatty acid chain will not just allow the removal of the matrix components but the penetration on the cell membrane with a bactericidal effect. Nevertheless, further work is required to confirm this hypothesis, taking into account that the effect in most of the cases would be species-specific. In addition, it will be worth to evaluate the combination between rhamnolipids and proteins, or any other molecule that could lead to find a specific strategy to eradicate biofilms of different microorganisms, either on static or continuous systems.
